# Low-Temperature Pyrolysis of PFOS-Contaminated Soil Enhanced by Additives: Thermodynamic Insights, Transformation Products, and Remediation Implications

**DOI:** 10.3390/toxics14060465

**Published:** 2026-05-26

**Authors:** Meichen Yao, Xiaodong Li, Chunhong Liu, Yayun Xiang, Jialun Shen, Lingjian Kong, Zongquan Sun, Dongsheng Zhang, Fujun Ma, Qingbao Gu, Boyan Gu

**Affiliations:** 1 State Key Laboratory of Environmental Criteria and Risk Assessment, Chinese Research Academy of Environmental Sciences, Beijing 100012, China; 2 State Key Laboratory of Pollution Control and Resources Reuse, College of Environmental Science and Engineering, Tongji University, Shanghai 200092, China; 3 Institute of Resources and Environment, Beijing Academy of Science and Technology, Beijing 100089, China; 4 College of Environmental Science and Engineering, Liaoning Technical University, Fuxin 123000, China

**Keywords:** enhancing pyrolysis, PFOS, additives, thermodynamic analysis, decomposition mechanism, economic cost

## Abstract

Perfluorooctanesulfonate (PFOS) is a persistent pollutant in soils due to its exceptional chemical and biological stability. Pyrolysis has been recognized as an effective technology for the remediation of PFOS-contaminated soil. However, its large-scale application faces challenges such as the requirement of high temperatures, long residence time, and corrosive off-gas treatment. The application of additives during pyrolysis is a promising strategy to overcome these challenges. In this study, six additives (Fe_2_O_3_, Fe_3_O_4_, CaO, Ca(OH)_2_, kaolinite, and MgO) were employed to improve PFOS removal from soil by pyrolysis. The effects of temperature, residence time, and removal efficiency with additives on the PFOS decomposition mechanism and economic benefits were systematically investigated. The results showed that all additives could allow for effective PFOS removal at a relatively low temperature (350 °C) and with a short residence time (30 min). Fe_2_O_3_ and CaO at a 5% dosage exhibited PFOS removal efficiency reaching 95.19% and 95.49%, respectively, which were 21.00% higher than that of the no-additive system. The thermodynamic analysis showed that the additives could reduce the activation energy (*Ea*) of PFOS pyrolysis, among which Fe_2_O_3_ showed the most significant effect (54.24 kJ/mol). Although additives exerted no significant effect on the type of PFOS decomposition products in soil, they effectively reduced the emission of acidic off-gases. Among them, CaO and Ca(OH)_2_ showed the most significant reduction by forming inorganic fluorides, followed by Fe_2_O_3_ and Fe_3_O_4_, through providing active sites. Economic analysis indicated that CaO had the lowest cost for PFOS removal (2.86 CNY/mg), followed by Fe_2_O_3_ (2.88 CNY/mg). Comprehensively considering PFOS removal efficiency, decomposition mechanism, economic cost, and pH of treated soil, Fe_2_O_3_ was identified as the optimal additive. This study provides new insights into the PFOS pyrolysis in soils, and proposes an energy-efficient remediation approach by reducing temperature, residence time, *Ea*, and off-gas emissions, which offers support for the large-scale application of this technology.

## 1. Introduction

Per- and polyfluoroalkyl substances (PFASs) are a class of anthropogenic chemicals with part or all the hydrogen atoms replaced by fluorine atoms on the carbon skeleton and with a terminal functional group [1,2]. Due to their strong C–F bond energy (536 kJ/mol) and amphiphilic nature, PFASs have been applied across multiple industries worldwide and are widely present in consumer products, such as food packaging, carpets, non-stick cookware, and fire-fighting foams, etc. [3,4]. Perfluorooctanesulfonate (PFOS) is a representative PFAS, consisting of a C8 fluorocarbon with a sulfonic acid functional group. Due to its extreme chemical and biological stability [1,5,6,7,8], PFOS was listed as a persistent organic pollutant in the Stockholm Convention in 2009 [9,10]. To date, PFOS has been widely detected in soil, which affects ecosystems and human health [11,12,13]. Toxicological studies have demonstrated that PFOS induced hepatotoxicity, thyroid disruption, immunotoxicity, reproductive and developmental toxicity in mammals and humans [14,15,16]. Moreover, the pyrolysis decomposition products of PFOS inevitably generated short-chain PFAS transformation products. Although their toxicological profiles remain incompletely characterized, these products could still exert immunotoxic effects [17]. Current studies indicated that the acute toxicity for thermal degradation products was lower than that of the parent PFASs, but they still posed potential risks to human health [18,19]. Meanwhile, PFOS concentrations in contaminated soils remain concerningly high. Research has shown that PFOS concentrations in soils from a U.S. metropolitan area remained as high as 157.8 μg/kg even ten years after the cessation of its production [20]. In Hubei Province, China, PFOS concentration ranged from 392.0 to 7.9 × 10^5^ μg/kg in a typical former site for PFOS production [21]. However, relatively limited treatment technology has been given to address PFOS in soil, despite high PFOS being frequently detected in contaminated sites.

Currently, the approaches for treating PFOS-contaminated soils include soil washing, immobilization, and pyrolysis [22,23,24]. Soil washing involves the use of chemical agents, which causes significant soil disturbance and poses a risk of secondary pollution [25]. Immobilization reduces the mobility and bioavailability of PFOS through sorption, but it does not destroy the molecular structure of PFOS [26]. Pyrolysis has emerged as a promising strategy for the remediation of PFOS-contaminated soils because elevated temperatures can effectively destruct and remove the parent PFOS [27]. However, an off-gas treatment system is essential to prevent secondary pollution from fluorinated byproducts [28]. Previous studies have shown that PFOS in contaminated soil could be substantially destroyed (>99%) at 500 °C in 30 min, whether in air or nitrogen atmosphere [23]. Although pyrolysis has demonstrated effectiveness in removing PFOS from soil, its application faces limitations due to the requirement of high temperatures, long residence time, and corrosive off-gas treatment. Therefore, achieving efficient removal of PFOS from soil with low energy consumption and mitigating the environmental risks of corrosive off-gas are challenges for promoting the broader application of pyrolysis.

The additives have recently emerged as a promising strategy to address the limitations of pyrolysis for polluted soil [29,30]. Liu et al. demonstrated that the addition of Fe_2_O_3_, Al_2_O_3_, and lime during pyrolysis of petroleum-contaminated soils significantly reduced the activation energy (*Ea*), consequently enhancing removal efficiency by up to 25.5% [31]. Abou-Khalil et al. demonstrated that lime-assisted heating achieved up to 95% PFOS removal at <500 °C, whereas removal without additives remained below 50% even at 800 °C [32]. However, few studies have investigated additive-assisted PFOS pyrolysis in soil. Consequently, the mechanism of additives in enhancing PFOS removal remains unclear, and thermodynamic insights into PFOS pyrolysis in soil are still limited.

In this study, the effects of temperature and residence time on PFOS removal during soil pyrolysis were systematically investigated in the presence of six additives (Fe_2_O_3_, Fe_3_O_4_, CaO, Ca(OH)_2_, kaolinite, and MgO). The *Ea* and thermodynamic parameters associated with additive-assisted pyrolysis were determined. Meanwhile, the pyrolysis products and off-gas compositions of PFOS were comparatively analyzed. The energy consumption and economic performance of the additives were further evaluated to identify the most favorable additive. The results provide new insights into the pyrolysis of PFOS-contaminated soils and propose an energy-efficient and safe remediation strategy, supporting the large-scale application of pyrolysis technology.

## 2. Materials and Methods

### 2.1. PFOS Chemicals and Soil Sample Preparation

PFOS and the isotope-labeled compound were provided by J&K Scientific Ltd. (Beijing, China) and Wellington Laboratories Inc. (Guelph, ON, Canada). Background soil from the Shenyang region without PFOS was used as the matrix soil. The physicochemical properties of the soil were shown in Table S1. A PFOS stock solution (0.5 mg/mL in methanol) was diluted with deionized water, and the diluted mixture was gradually spiked into 1 kg of clean soil in a plastic container under continuous stirring to achieve a target PFOS concentration of 10 mg/kg. After thorough blending, the container was placed on a turnover oscillator for 48 h to achieve homogeneous PFOS distribution in the soil matrix. The homogenization procedure was considered complete when the relative standard deviation (RSD) of PFOS concentrations in triplicate subsamples was below 5%. Afterwards, the container was unsealed and transferred to a fume hood to allow complete evaporation of the solvent. The soil was subsequently stored in the dark for a six-month aging period. The extraction and analysis of PFOS in soil were conducted following previously established procedures [33].

### 2.2. Pyrolysis Experiments

A total of 15.0 g of contaminated soil was thoroughly mixed with additives (Fe_2_O_3_, Fe_3_O_4_, CaO, Ca(OH)_2_, kaolinite, and MgO) at different weight percentages (0%, 1%, 2%, and 5%), and then subjected to thermal treatment in a tube furnace. A continuous nitrogen flow of 1 L/min was applied to prevent HF deposition on the furnace. Based on previous studies, the heating temperature of 350 °C, residence time of 30 min, and soil moisture content of 10% were selected as the heating experiment conditions [32]. After each run, the treated soil was allowed to cool naturally to room temperature under gas flow conditions. The treated soil samples were stored in a refrigerator at 4 °C for further analysis. Each experiment was conducted in triplicate. In this study, PFOS removal efficiency was calculated as the percentage reduction in extractable PFOS concentration from soil after pyrolysis, relative to the initial concentration.

### 2.3. Thermogravimetric and Thermodynamic Parameter Analysis

A thermogravimetric (TG) analyzer (Netzsch, STA449F3, Selb, Germany) was used to conduct thermogravimetric experiments on soil samples with additives. The inert atmosphere was maintained by the continuous flow of argon gas. In non-isothermal experiments, each soil sample was heated from 30 °C to 800 °C at heating rates of 5 °C/min, 10 °C/min, and 20 °C/min. The reaction rate of the sample during the heating can be expressed as follows [34]:

(1) $$\frac{d\alpha }{dt}=Aexp\left(-\frac{E_{a}}{RT}\right)f\left(\alpha \right)$$

where *α* represents the conversion rate; *t* is the reaction time (min); *A* refers to the pre-factor (1/s); *Ea* is the activation energy (J/mol); *R* is the universal gas constant (*R* = 8.3145 J/(mol·K)); *T* stands for absolute temperature (K); *f*(*α*) represents the differential form of the reaction model.

The heating rate *β* = d*T*/d*t* is introduced into Equation (1) to yield Equations (2) and (3).

(2) $$\frac{d\alpha }{dT}=\frac{A}{\beta }exp\left(-\frac{E_{a}}{RT}\right)f(\alpha )$$

(3) $$g\left(\alpha \right)=\int_{0}^{\alpha }\frac{d\alpha }{f\left(\alpha \right)}=\frac{A}{\beta }\int_{0}^{T}exp\left(-\frac{E_{a}}{RT}\right)dT$$

where *g*(*α*) is the integral function of the conversion rate *α*. 

The Flynn–Wall–Ozawa (FWO) method was used to calculate the *Ea*, expressed by Equation (4), where *Ea* is determined by plotting the relationship between In*β* and 1/*T*.

(4) $$In\left(\beta \right)=ln\frac{AE_{a}}{g(\alpha )R}-5.331-1.052\left(\frac{E_{a}}{RT}\right)$$

The thermodynamic parameters (including *Ea*, ∆*H*, ∆*G*, and ∆*S*) of heating were calculated with the FWO method, which is based on the Arrhenius equations [35,36].

(5) A=βEaexp(EaRTpeak)RTpeak2

(6) $$∆H=E_{a}-RT$$

(7) $$∆G=E_{a}+RT_{peak}ln\left(\frac{K_{B}T_{peak}}{hA}\right)$$

(8) $$∆S=\frac{∆H-∆G}{T_{peak}}$$

where *T_peak_* is the peak temperature of the DTG curve, *K_B_* is the Boltzmann constant (1.381 × 10^−23^ J/K), and *h* is Plank’s constant (6.626 × 10^−34^ J·s).

### 2.4. Analysis Method for PFOS Decomposition Products

The PFOS decomposition products analysis was performed using an ultra-high-performance liquid chromatography coupled with a high-resolution Orbitrap mass spectrometry system (UHPLC-Orbitrap HRMS). Chromatographic separation was achieved on an ACQUITY UPLC CSH C18 column (100 mm × 2.1 mm, 1.7 µm; Waters, Milford, MA, USA) maintained at 40 °C with a flow rate of 300 µL/min. Mobile phase A consisted of 5 mM ammonium acetate in methanol–water (10:90, *v*/*v*), and mobile phase B was 5 mM ammonium acetate in methanol. The gradient elution program was shown in Table S2.

Mass spectrometric analysis was conducted in both positive and negative ion modes with a scan range of 80–1000 *m*/*z*. Positive ion mode parameters included a spray voltage of 3500 V, ion transfer tube temperature of 150 °C, vaporizer temperature of 200 °C, sheath gas flow rate of 50 arbitrary units, and stepped HCD collision energies of 20%, 40%, and 60%. Negative ion mode parameters were identical except for a spray voltage of 1000 V. Both full-scan MS and MS/MS analyses were performed at resolutions of 60,000 and 15,000, respectively.

### 2.5. Fourier Transform Infrared Spectroscopy (FTIR) Analysis of Products in Off-Gas

The off-gas from PFOS-contaminated soil with different additives by pyrolysis was analyzed using FTIR. The contaminated soil samples were heated at a rate of 10 °C/min from 30 °C to 800 °C, and the FTIR spectra of the off-gas were recorded from 400 to 4000 cm^−1^ per sample. The background signal was collected and subtracted before each experiment. In addition, in order to ensure the gas products were completely expelled, argon was kept flowing for 30 min after each experiment.

## 3. Results and Discussion

### 3.1. Enhancing Effect of Additives on PFOS Pyrolysis in Soil

**Figure 1:**
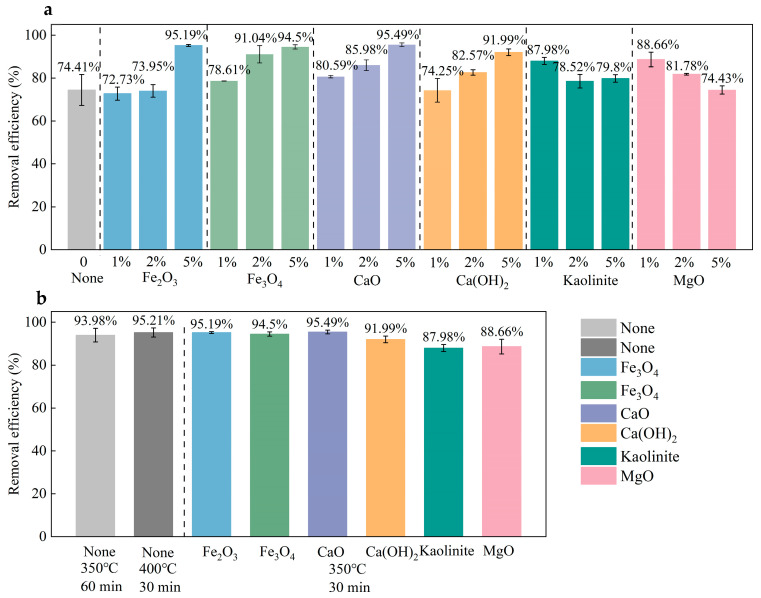
Removal efficiency of PFOS-contaminated soil under different conditions. (**a**) Removal efficiency of PFOS with various additives at 350 °C for 30 min, and (**b**) removal efficiency of PFOS with additives at different temperatures and residence times.

Compared with no additive, the parent PFOS removal efficiency reached 72.73–95.49% at 350 °C within 30 min with various additives (Figure 1a). The PFOS removal efficiency improved gradually with the increasing dosage of Fe_2_O_3_, Fe_3_O_4_, Ca(OH)_2_, and CaO. When the addition of Fe_2_O_3_ and Fe_3_O_4_ increased from 1% to 5%, PFOS removal efficiency rose from 72.73% to 95.19%, and 78.61% to 94.50%, respectively. The improved removal efficiency may be partly attributed to the role of Fe_2_O_3_ and Fe_3_O_4_ as thermal carriers, which could enhance heat transfer and promote more uniform temperature distribution within the soil, thereby reducing the risk of localized underheating [37]. In addition, a catalytic contribution from the iron oxides cannot be ruled out. The addition of 5% CaO significantly enhanced the removal efficiency of PFOS to 95.49%. Due to the chemical similarity between Ca(OH)_2_ and CaO, 5% Ca(OH)_2_ exhibited over 90% removal efficiency. The enhancement by CaO and Ca(OH)_2_ is mainly attributed to alkaline environment regulation, significantly facilitating PFOS reaction and accelerating C–F bond cleavage. Adding 1% kaolinite or MgO could enhance the removal efficiency by approximately 15%. However, the PFOS removal efficiency gradually decreased with increasing dosages of these two additives. Kaolinite adsorbed and enriched PFOS molecules around its active sites via its large specific surface area, thereby enhancing PFOS removal efficiency [38]. Kaolinite exhibits a low effective thermal conductivity (less than 0.3 W·m^−1^·K^−1^) [39]. Accordingly, excess kaolinite particles encapsulated soil aggregates and formed a thermal barrier, which impeded heat transfer and consequently reduced the PFOS removal efficiency. For MgO, the reduction in efficiency at higher loadings may be attributed to particle agglomeration that shields catalytically active defect sites [40]. These results clearly demonstrated that kaolinite and MgO can only be used at low dosages.

Figure 1b compares the maximum PFOS removal efficiencies with and without additives at different temperatures and residence times. The results demonstrated that the PFOS removal efficiency reached 93.98% at 350 °C for 60 min or 95.21% at 400 °C for 30 min. These values were comparable to the removal efficiencies achieved with Fe_2_O_3_, Fe_3_O_4_, and CaO at 350 °C for only 30 min, which reached 95.19%, 94.50%, and 95.49%, respectively. These results indicated that adding additives can reduce the temperature for PFOS removal by 50 °C and shorten the residence time by 50%, thereby significantly lowering the energy consumption of pyrolysis.

### 3.2. Thermogravimetric and Thermodynamic Parameter of Different Additives

**Figure 2:**
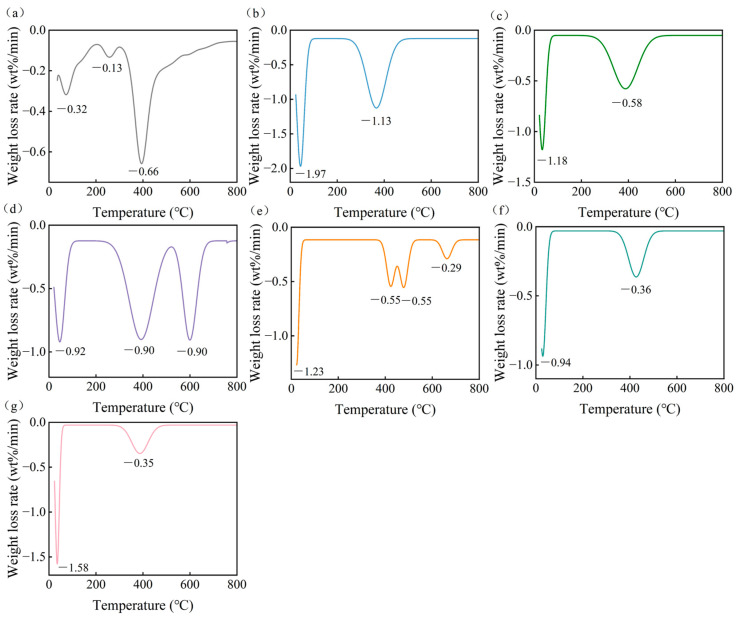
DTG of adding different additives in PFOS-contaminated soil. (**a**) No additives, (**b**) Fe_2_O_3_, (**c**) Fe_3_O_4_, (**d**) CaO, (**e**) Ca(OH)_2_, (**f**) kaolinite, and (**g**) MgO.

The TG and thermogravimetric (DTG) curves of PFOS-contaminated soils without and with additives were shown in Figure S1 and Figure 2, respectively. As shown in Figure 2, the curves for Fe_2_O_3_, Fe_3_O_4_, CaO, and Ca(OH)_2_ exhibited higher peak intensity, indicating that these additives accelerated the PFOS decomposition rate. The low DTG peak intensity of kaolinite and MgO reflected a negligible enhancement of PFOS decomposition rate, consistent with the results of removal efficiency. Correspondingly, these variations in pyrolysis behavior further demonstrated that different additives can markedly alter the pyrolysis process of PFOS in contaminated soil.

Without additives, the pyrolysis process of PFOS-contaminated soil could be divided into two decomposition stages: I (200–300 °C), and II (300–800 °C). Fe_2_O_3_ and Fe_3_O_4_ catalytically merged PFOS decomposition into stage I (200–800 °C). When CaO was added, the thermal process still included two decomposition stages, but with a modified temperature range: I (200–600 °C), and II (600–800 °C). Ca(OH)_2_ showed wider DTG peaks and more decomposition stages (I: 200–450 °C, II: 450–550 °C, and III: 550–800 °C) resulting from its catalysis and fluorine immobilization. The pyrolysis process was dominated by water removal below 200 °C [41].

**Figure 3:**
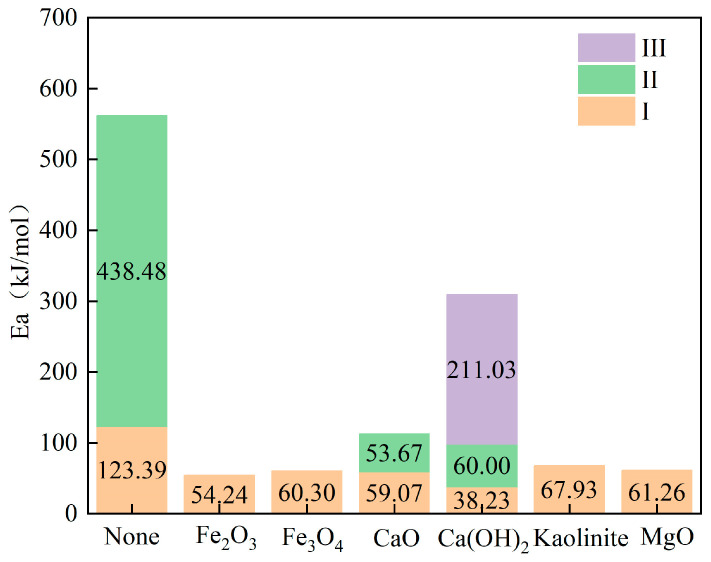
*Ea* values for PFOS-contaminated soil with and without additives.

To further elucidate the removal mechanism of PFOS induced by additives, the *Ea* of each pyrolysis stage was calculated (Figure 3). It was found that all additives decreased the *Ea*, indicating that additives reduced the energy barrier for PFOS pyrolysis. Stage II for PFOS removal without additives exhibited *Ea* as high as 438.48 kJ/mol, making it the core rate-limiting step and the root cause of the high energy consumption in pyrolysis. The addition of Fe_2_O_3_ and Fe_3_O_4_ reduced *Ea* of the core PFOS pyrolysis stage by more than 85% via the catalytic effect, which enabled efficient PFOS removal at low temperature. Accordingly, the lowest *Ea* (54.24 kJ/mol) was achieved with the addition of Fe_2_O_3_. Although the total *Ea* of CaO and Ca(OH)_2_ were relatively high, they were still far lower than that of the core rate-limiting step without additives. This was because CaO and Ca(OH)_2_ greatly reduced the initial *Ea* of PFOS pyrolysis (stage I) via the effects of alkaline catalysis and fluorine immobilization. At the same time, the rate-limiting step was divided into two consecutive reactions with low energy barriers, thereby still achieving an improvement in PFOS removal efficiency.

The thermodynamic parameters (Δ*H*, Δ*G*, and Δ*S*) for PFOS pyrolysis in soil with different additives were determined from the FWO fitting plots shown in Table 1 and Figure S2. Based on Equations (1)–(8) presented in Section 2.3, linear fitting was performed by plotting ln*β* against 1/*T*. The thermodynamic parameters were further determined according to the slope of fitted straight lines. The Δ*H* values exhibited a similar trend to *Ea* with comparable numerical values. The decrease in Δ*H* values with additives showed that energy requirement for PFOS removal was reduced, which thereby facilitated the pyrolysis process [34,42]. The positive Δ*G* values suggest that PFOS pyrolysis is non-spontaneous and requires external energy input, reflecting the complexity of the reaction [34]. The Δ*S* represents the disorder degree of the pyrolysis system, with larger absolute values corresponding to greater system stability [43]. In this study, the Δ*H*, Δ*G*, and Δ*S* were decreased with the addition of Fe_2_O_3_, Fe_3_O_4_, kaolinite, and MgO. The Δ*H* of core pyrolysis stage I was reduced by over 50% with the addition of Fe_2_O_3_ (53.10 kJ/mol) and Fe_3_O_4_ (58.93 kJ/mol), compared with 129.58 kJ/mol for the same stage without additives. The decreased Δ*S* was attributed to the immediate adsorption and continuous decomposition of gaseous products and intermediates on active sites, which reduced system disorder via the Fe^2+^/Fe^3+^ redox cycle [44]. Kaolinite and MgO showed the same trend in thermodynamic parameters as Fe_2_O_3_ and Fe_3_O_4_, due to the enhanced PFOS adsorption by the layered structure of kaolinite and the PFOS decomposition promoted by the surface defect sites of MgO. In contrast, CaO and Ca(OH)_2_ increased Δ*H* and Δ*G*, consistent with the trend of *Ea*. The significant decrease in Δ*S* for CaO and Ca(OH)_2_ was ascribed to the adsorption of intermediates on alkaline active sites and immobilization of products (CaF_2_, CaCO_3_) in the solid phase, which reduced system disorder (Figure S3) [44,45]. These results demonstrated that additives accelerated PFOS decomposition and facilitated the formation of more ordered products [34].

### 3.3. Influence of Additives on Decomposition Products

#### 3.3.1. Decomposition Products in Soil

**Figure 4:**
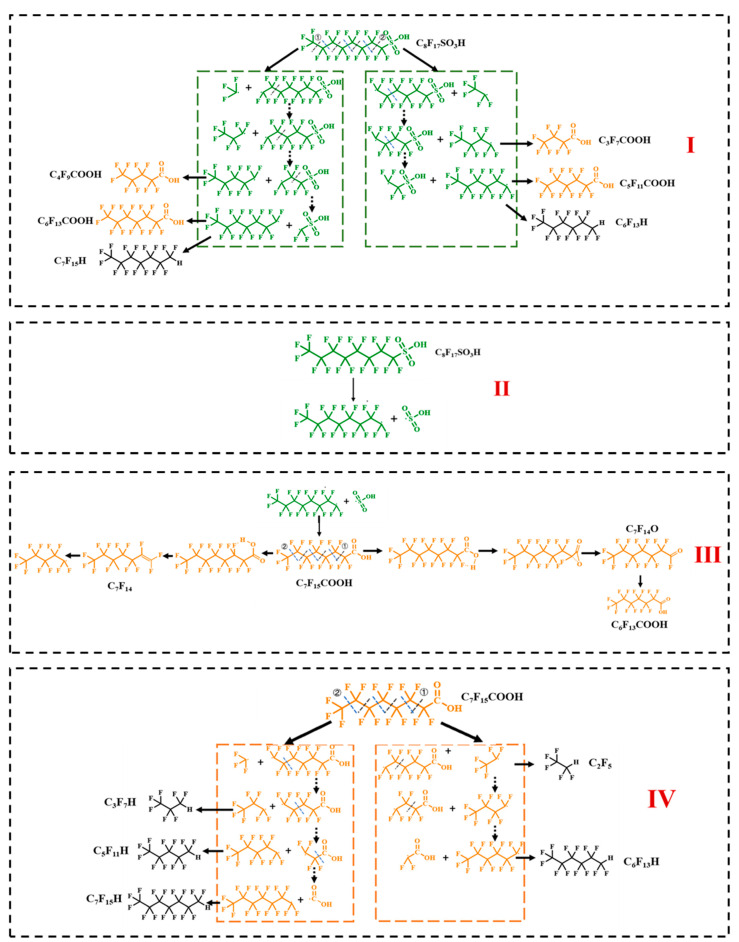
Decomposition pathway of PFOS with additives.

The decomposition products and pathways of PFOS with additives were shown in Figure 4 and Table S3. PFOS was partially decomposed in four stages during pyrolysis with 14 decomposition products, including short-chain perfluorocarboxylic acids, short-chain perfluoroalkenes, and other perfluoroalkyl compounds. It was reported that the formation of these products mainly derived from short-chain perfluoroalkyl radicals produced by the random cleavage of C–C and C–S bonds, and then further decomposed via β-scission, hydration, defluorination, and dehydration reactions [46]. C–S bond cleavage caused the dissociation of the sulfonic acid functional group, forming reactive radicals including C_8_F_17_^•^, SO_3_^•−^, C_8_F_17_^+^, and SO_3_^2−^ [47]. Although these radicals were not directly observed, their formation was inferred based on the detection of perfluorocarboxylic acids and perfluoroalkenes. For instance, C_8_F_17_^•^ was recognized as a highly reactive intermediate. On one hand, the unpaired electron on the α-carbon transferred to the adjacent β-carbon, polarizing the C–C bond and inducing heterolytic cleavage of C–F bonds. This process generated short-chain perfluoroalkyl radicals, such as C_6_F_13_^•^ and C_4_F_9_^•^, along with perfluoroalkenes. On the other hand, C_8_F_17_^•^ reacted with inherent oxygen sources, such as lattice oxygen, bound water, or oxygen-containing functional groups in the soil matrix or additives, to form acyl fluoride radicals C_7_F_15_C(O)F, which further transformed into PFOA [48]. SO_3_^•−^, with its strong oxidative properties, was another proposed intermediate, which was presumed to abstract a hydrogen atom from the soil matrix to form the bisulfite radical (HSO_3_^•^) [19]. At high temperatures, the S–O bond in HSO_3_^•^ was cleaved, releasing SO_2_ and ^•^OH radicals [48]. These active radicals facilitated the degradation of perfluoroalkenes into short-chain perfluorocarboxylic acids, which may further form hydrofluoro-substituted perfluorocarboxylic acids. The proton from an acidic functional group previously formed PFOA may interact with a nearby fluorine from the C–F backbone to form a five-or six- membered transition state. The five-membered transition state reacted with oxygen sources to form acyl fluoride radicals and short-chain perfluorocarboxylic acids. The six-membered transition state formed unsaturated perfluoroalkene intermediates and eventually hydro-substituted fluorocarbons [19]. The random C–C cleavage was similar to that in PFOS, generating hydrogen-substituted products. Simultaneously, analogous to the decomposition process of PFOS, the cleavage of the perfluorinated carbon chain of PFOA at random C–C bonds formed short-chain perfluoroalkyl radicals that were ultimately converted to perfluoroalkene products.

**Figure 5:**
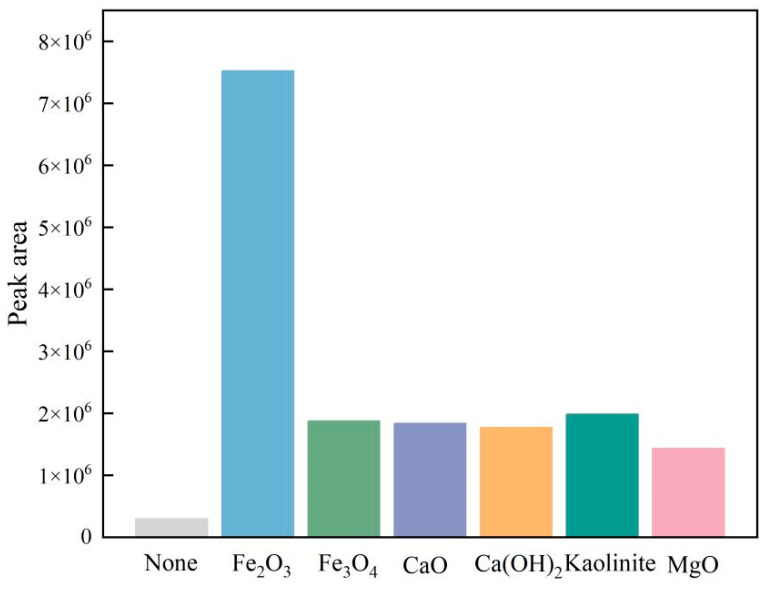
Yield of products with different additives.

Additive types significantly influenced the yield of decomposition products. Owing to the lack of standard samples for some reaction intermediates, peak area was used herein for the comparative assessment of product formation (Figure 5). The results showed that Fe_2_O_3_ significantly promoted the formation of all products, and the corresponding peak area was approximately four times that of other additives. This was followed by kaolinite, Fe_3_O_4_, CaO, Ca(OH)_2_, and MgO. Consistent with the thermodynamic analysis, Fe_2_O_3_ substantially decreased the *Ea* while simultaneously providing surface acidic sites under thermal conditions, which facilitated electron transfer and accelerated PFOS dehydrofluorination to form conjugated double bonds. Furthermore, the formation of Fe_3_O_4_ observed in XRD indicated that Fe_2_O_3_ not only acted as a catalyst but also participated in the reaction as an oxidant (Figure S3). The adsorption and surface hydroxyl-mediated catalysis of kaolinite could accelerate the formation and accumulation of organic fragments and products, leading to an increase in product yield [49,50,51,52]. After adding Fe_3_O_4_, a substantial amount of short-chain perfluoroalkyl compounds was generated, indicating its capacity to promote C–C bond cleavage in PFOS. The product yield of Fe_3_O_4_ was low because Fe_3_O_4_ is an efficient electron-mediated catalyst, with Fe^3+^ ions rapidly capturing electrons to facilitate the reaction, although its oxidizing power is lower than that of Fe_2_O_3_ [53]. The product yield was similar to that of Fe_3_O_4_ when CaO or Ca(OH)_2_ was added. CaO or Ca(OH)_2_ could convert part of the organic compounds into inorganic substances (such as CaF_2_ and CaCO_3_ in Figure S3), leading to a lower content of organic products in the soil.

#### 3.3.2. Gaseous Products

**Figure 6:**
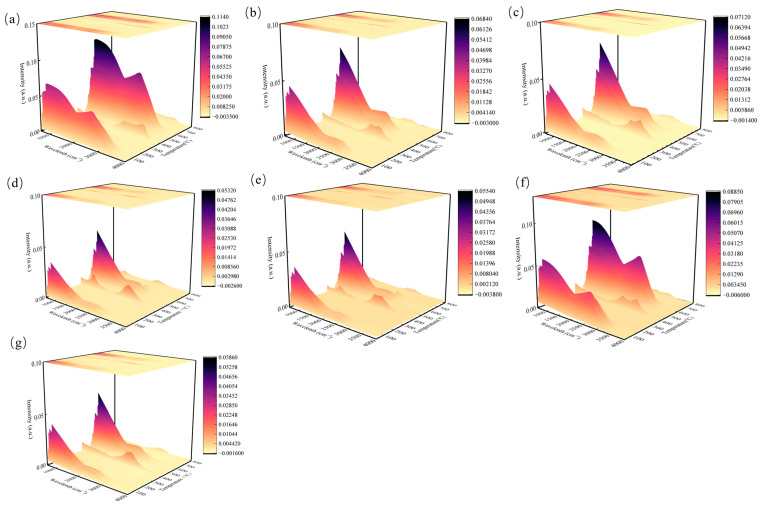
3D-FTIR of PFOS-contaminated soil heated without and with additives. (**a**) No additions, (**b**) Fe_2_O_3_, (**c**) Fe_3_O_4_, (**d**) CaO, (**e**) Ca(OH)_2_, (**f**) kaolinite, (**g**) MgO.

The gaseous products released during the heating of PFOS-contaminated soil were monitored in real time using a thermogravimetric analyzer coupled with Fourier-transform infrared spectroscopy. Figure 6 and Figure S4 showed the three-dimensional FTIR spectra and corresponding spectral curves obtained with and without additives. Various gaseous products, such as perfluorocarbons, perfluoroalkenes, HF, SO_2_, and CO_2_, were detected [54]. Signals ranging from 1000 to 1300 cm^−1^ corresponded to the vibration of C–F bonds. The band at 1350–1370 cm^−1^ were attributed to the asymmetric stretching of S=O stretching. The vibrational feature of HF emerged within 3500–4000 cm^−1^, while the absorption signals at 2240–2400 cm^−1^ derived from the asymmetric C=O stretching vibration of CO_2_. Previous studies found that C_8_F_16_O was initially formed when the temperature was above 500 °C, then substantial amounts of COF_2_ and C_2_F_4_ were generated. These intermediates can be identified by the C=O stretching vibration at 1940 cm^−1^, the asymmetric stretching of CF_2_ at 1300–1360 cm^−1^, and the symmetric stretching at 1150–1210 cm^−1^ [55,56]. In this study, the symmetric stretching of CF_2_ was not clearly detected, likely due to its relatively low concentration in soil, which suppressed the CF_2_ absorption peaks in the infrared spectra.

According to the Beer–Lambert law [57], the concentration of volatile products is positively correlated with the intensity of absorption peaks. Figure S4 demonstrated that infrared peak intensities increased with temperature, reflecting more gaseous products formed during PFOS pyrolysis. Compared with the group without additives, all additives significantly reduced the gaseous product yield of PFOS at the same temperature. At temperatures above 700 °C, the accelerated decomposition of C_n_F_m_ species led to the enhanced formation of CF_2_ radicals, which subsequently reacted with water vapor and hydroxyl groups to produce increased amounts of HF [55]. CF_2_ radicals will also trigger recombination reactions, resulting in the formation of short-chain perfluoroalkanes and perfluoroalkenes. Meanwhile, CF_2_ radicals could adsorb onto the metal surface, which explained the reason why FeF_2_, FeF_3_, and MgF_2_ were formed after adding Fe_2_O_3_, Fe_3_O_4_, and MgO [58].

The FTIR peaks of gaseous products were integrated to quantify the suppression effects of each additive on off-gas emissions (Table S4). The peak area reduced most markedly with CaO, followed by Fe_3_O_4_, MgO, Ca(OH)_2_, and Fe_2_O_3_, whereas the weakest reduction in peak area was observed for kaolinite. This trend was consistent with the 3D-FTIR spectrums in Figure 6. This was attributed to the adsorption of kaolinite, which reduced the apparent yield of F by adsorbing PFOS in soil and F radicals in the off-gas. However, such adsorption did not significantly alter the parent PFOS removal efficiency or the formation of gaseous products [23]. Fournie et al. [59] have shown that alkaline earth metal compounds can promote the formation of metal fluoride precipitates, effectively suppressing the generation of various gases (especially acidic gases such as SO_2_ and HF), which alleviates the difficulty of off-gas treatment. H_2_O and CF_3_ radicals are absorbed on iron metal composite sites to form FeOH_2_ [60], which facilitates the formation of FeF_3_ and SO_2_. In contrast, kaolinite exposed abundant Lewis acid sites (Al^3+^) after dehydroxylation, which strongly polarized C–F bond to produce HF, with potential implications for subsequent processing [61]. Major gas products, such as H_2_O, CO_2_, HF, and COF_2_, containing OH, C-O, and C=O groups, primarily arose from the dehydrogenation, deoxygenation, and defluorination of PFOS and other oxygen-containing groups in the soil, as well as the decomposition of soil minerals [62]. Between 300 and 500 °C, PFOS underwent minor decomposition to CO_2_, while soil organic matter degraded extensively into CO_2_, leading to increased infrared peak intensity. At 500–800 °C, PFOS decomposed on a large scale, predominantly to SO_2_ and fluorocarbons through radical-mediated pathways, causing a decline in CO_2_ intensity. When the temperature was above 800 °C, oxygen species released from PFOS decomposition reacted with the CF_2_ radical to form additional CO_2_ [63], while the intensities of other infrared peaks increased with temperature.

### 3.4. Energy Consumption and Economic Benefits for Pyrolysis

The energy consumption per unit mass of removed PFOS by pyrolysis with different additives is shown in Table 2. The energy-saving effect was related to the type and dosage of additives. As the additive dosage increased, the energy consumption for PFOS removal decreased for Fe_2_O_3_, Fe_3_O_4_, CaO, and Ca(OH)_2_, but increased for kaolinite and MgO. Among the six additives, CaO and Fe_2_O_3_ exhibited the lowest energy consumption for PFOS removal (3.97 and 3.98 kW·h/mg). Compared with no additives, the energy consumption was reduced by 22.00% and 21.81% for Fe_2_O_3_ and CaO, respectively, indicating that the addition of Fe_2_O_3_ and CaO can effectively reduce the reaction energy consumption. In contrast to the other additives, kaolinite and MgO exhibited an opposite trend in energy consumption. Only at a low dosage of 1% was a slight energy-saving effect observed. The energy consumption continuously increased with increasing dosage. These findings were consistent with the removal efficiency results.

According to the prices of additives (Table S5) and the industrial electricity (0.721 CNY/kW·h), the cost per unit mass of removed PFOS by pyrolysis was calculated for each additive (Table 2). The cost was 3.67 CNY/mg with no additives. After the addition of additives, the lowest removal cost per unit mass of PFOS was 2.86 CNY/mg (CaO), followed by 2.88 CNY/mg (Fe_2_O_3_), which was consistent with the energy consumption results. The lowest cost with additives (2.86 CNY/mg for CaO) was 22.07% lower than that without additives. Therefore, based on the evaluation of energy consumption and cost, CaO was the most energy-efficient additive, followed by Fe_2_O_3_.

In summary, both CaO and Fe_2_O_3_ could achieve PFOS removal efficiency of more than 95%, followed by Fe_3_O_4_, Ca(OH)_2_, MgO, and kaolinite. In terms of thermodynamic performance, Fe_2_O_3_ had the lowest *Ea* value (54.24 kJ/mol) in the pyrolysis process, succeeded by MgO, kaolinite, Fe_3_O_4_, CaO, and Ca(OH)_2_. Concerning the decomposition products, all additives had no significant influence on the types of products. However, the addition of Fe_2_O_3_ could promote product yield. Nevertheless, five additives except kaolinite could suppress the production of acidic off-gas, among which CaO and Ca(OH)_2_ displayed the most remarkable reduction, followed by MgO, Fe_2_O_3_, and Fe_3_O_4_. With respect to energy consumption and economic benefits, the cost remained below 3 CNY/mg when 5% Fe_2_O_3_, Fe_3_O_4_, or CaO was added, among which CaO (2.86 CNY/mg) presented the lowest cost, followed by Fe_2_O_3_ (2.88 CNY/mg) and Fe_3_O_4_ (2.90 CNY/mg). Therefore, based on the analysis of removal efficiency, acidic off-gas emissions and cost, CaO may be the most suitable additive. From a toxicity-weighted perspective, CaO showed excellent performance in suppressing acidic off-gases, thereby mitigating the associated acute toxicity and corrosive risks. However, its application raised soil pH to 11–12, which can severely influence soil microbial communities and plant growth [64]. Furthermore, such strongly alkaline conditions may alter the speciation of heavy metals, potentially inducing secondary toxicity risks [65]. In contrast, Fe_2_O_3_ is a natural mineral and its exogenous addition imposed negligible disturbance on soil physicochemical properties. It effectively reduced the *Ea* of PFOS pyrolysis and achieved a high PFOS removal efficiency. Although its capacity to suppress acidic off-gases was weaker than that of CaO, Fe_2_O_3_ caused less damage to soil ecological functions [66]. Moreover, animal carcinogenicity studies have demonstrated that Fe_2_O_3_ exhibited no carcinogenic potential, whereas the median lethal dose (LD_50_) of CaO was 3059 mg/kg (intraperitoneal, mouse), as recorded in the National Institute for Occupational Safety and Health (NIOSH) Toxicity database. Accordingly, Fe_2_O_3_ presents substantially lower long-term ecological toxicity risks to soil than CaO. Overall, comprehensively considering PFOS removal efficiency, soil pH, toxicity and economic cost, 5% Fe_2_O_3_ was identified as the optimal additive.

## 4. Conclusions

This study investigated the enhancement effects and underlying mechanisms of six additives on the pyrolysis of PFOS-contaminated soil. The results demonstrated that additives could reduce PFOS pyrolysis temperature and residence time, while enhancing the PFOS removal. 5% Fe_2_O_3_ and CaO showed the most significant enhancement in PFOS removal efficiency (exceeding 95%), which was 21% higher than that without additives. All additives reduced the *Ea* values of PFOS pyrolysis, with Fe_2_O_3_ showing the greatest reduction (54.24 kJ/mol). The decomposition of PFOS with additives contained four stages, including random chain scission of carbon of PFOS, scission of the functional group and PFOA formation, HF elimination through a five- or six-membered transition state, and random chain scission of carbon of PFOA. The addition of Fe_2_O_3_ increased the product yield significantly while promoting PFOS decomposition. Except for kaolinite, all other additives reduced emissions of acidic off-gas (e.g., HF, SO_2_) via the formation of inorganic fluorides (CaF_2_, FeF_3_, MgF_2_), with CaO and Ca(OH)_2_ showing the greatest reduction, followed by Fe_2_O_3_ and Fe_3_O_4_. Economic analysis indicated that CaO had the lowest cost for PFOS removal (2.86 CNY/mg), followed by Fe_2_O_3_ (2.88 CNY/mg). Although CaO was highly effective in suppressing acidic off-gas emissions and exhibited the lowest cost, its addition to soil can markedly increase soil pH, thereby limiting subsequent soil reuse. Therefore, Fe_2_O_3_ was selected as the most suitable additive in this study. Nevertheless, CaO may be preferable where acidic off-gas suppression is the dominant operational priority and post-treatment soil reuse is not required. This study provided a practical and sustainable strategy for the thermal remediation of PFOS-contaminated soils.

## Figures and Tables

**Table 1:** The Δ*H*, Δ*G*, Δ*S* variation trends of PFOS-contaminated soil without and with additives at thermal decomposition stages.

Type	Stage	Δ*H* (kJ/mol)	Δ*G* (kJ/mol)	Δ*S* (J/mol)
None	I	129.58	63.59	247.52
	II	159.79	92.59	−15.68
Fe_2_O_3_	I	53.10	125.15	−149.52
Fe_3_O_4_	I	58.93	120.18	−116.18
CaO	I	55.95	129.23	−147.19
	II	46.11	190.33	−204.20
Ca(OH)_2_	I	34.73	112.11	−179.27
	II	52.43	118.70	−144.05
	III	147.60	120.96	55.28
Kaolinite	I	60.14	122.93	−138.63
MgO	I	62.33	126.99	−141.37

**Table 2:** Energy consumption per unit mass of PFOS removal with different additives.

Additives	Dosage	Energy Consumption (kW·h/mg)	Cost (CNY/mg)
None	0	5.09	3.67
Fe_2_O_3_	1%	5.21	3.76
	2%	5.12	3.70
	5%	3.98	2.88
Fe_3_O_4_	1%	4.82	3.48
	2%	4.02	2.91
	5%	4.01	2.90
CaO	1%	4.70	3.39
	2%	4.41	3.18
	5%	3.97	2.86
Ca(OH)_2_	1%	5.10	3.68
	2%	4.59	3.31
	5%	4.12	2.97
Kaolinite	1%	4.27	3.08
	2%	4.63	3.34
	5%	5.09	3.68
MgO	1%	4.31	3.11
	2%	4.83	3.49
	5%	4.75	3.44

## Data Availability

The original contributions presented in this study are included in the article/Supplementary Materials. Further inquiries can be directed to the corresponding author(s).

## Supplementary Materials

The following supporting information can be downloaded at https://www.mdpi.com/article/10.3390/toxics14060465/s1. Figure S1: Thermal weight of adding different additives in PFOS-contaminated soil on different heating rates. (a) Fe_2_O_3_, (b) Fe_3_O_4_, (c) CaO, (d) Ca(OH)_2_, (e) kaolinite, (f) MgO, (g) No additions. Figure S2: Arrhenius plots based on FWO method. Figure S3: XRD diagram of PFOS-contaminated soil with different additives. (a) Fe_2_O_3_, (b) Fe_3_O_4_, (c) CaO, (d) Ca(OH)_2_, (e) Kaolinite, (f) MgO, (g) No additions. Black: before heating; blue: after heating. Figure S4: Chromatograms of thermal decomposition with and without additives. (a) No additions, (b) Fe_2_O_3_, (c) Fe_3_O_4_, (d) CaO, (e) Ca(OH)_2_, (f) kaolinite, (g) MgO. Figure S5: FTIR spectra of off-gas from thermal treatment of PFOS-contaminated soil. (a) Fe_2_O_3_, (b) Fe_3_O_4_, (c) CaO, (d) Ca(OH)_2_, (e) kaolinite, (f) MgO, (g) No additions. Table S1: Physicochemical properties of soil. Table S2: Gradient elution procedure of LC–MS. Table S3: Decomposition products of PFOS. Table S4: Integrated areas of FTIR characteristic peaks for off-gases with and without additives. Table S5: Price of additives used for contaminated soil pyrolysis.
