# Supplementary material for: Low-Temperature Pyrolysis of PFOS-Contaminated Soil Enhanced by Additives: Thermodynamic Insights, Transformation Products, and Remediation Implications
Source: Toxics. 2026 May 26;14(6):465. doi: 10.3390/toxics14060465 (PMC13306712; doi:10.3390/toxics14060465)
Supplement: Supplementary file 1 [file toxics-14-00465-s001.zip › toxics-4277530-supplementary.pdf]

Low-Temperature Pyrolysis of PFOS-Contaminated Soil Enhanced by Additives: Thermodynamic Insights, Transformation Products, and Remediation Implications

Meichen Yao [1,2], Xiaodong Li [1], Chunhong Liu [3], Yayun Xiang [1], Jialun Shen [1], Lingjian Kong [1], Zongquan Sun [1], Dongsheng Zhang [1], Fujun Ma [1,*], Qingbao Gu [1,2,*] and Boyan Gu [4]

[1] State Key Laboratory of Environmental Criteria and Risk Assessment, Chinese Research Academy of Environmental Sciences, Beijing 100012, China

[2] State Key Laboratory of Pollution Control and Resources Reuse, College of Environmental Science and Engineering, Tongji University, Shanghai 200092, China

[3] Institute of Resources and Environment, Beijing Academy of Science and Technology, Beijing 100089, China

[4] College of Environmental Science and Engineering, Liaoning Technical University, Fuxin 123000, China

* Correspondence: mafj@craes.org.cn (F.M.); guqb@craes.org.cn (Q.G.); Tel.: +86-10-84915233 (Q.G.)

Table S1 Physicochemical properties of the soil.

Clay (%)	Silt (%)	Sand (%)	pH	moisture-holding capacity (g/g)	OM (g/kg)	F (mg/kg)
14.91	43.66	40.23	7.28	0.28	6.95	7.38

Table S2 The gradient elution procedure of LC-MS.

Time (min)	A	B
0.00	95%	5%
1.00	95%	5%
9.50	1%	99%
12.50	1%	99%
12.60	95%	5%
15.00	95%	5%

Table S3 Decomposition products of PFOS.

Formula of products	m/z	Retention time （min）
$C_7F_{15}COOH$	412.9651	9.47
$C_6F_{13}COOH$	362.9693	9.01
$C_5F_{11}COOH$	312.9721	8.47
C_4F_9COOH	262.976	1.33
$C_6F_{12}SO_3H_2$	380.9460	8.09
$C_7F_{14}SO_3H_2$	430.9428	1.36
$C_5F_{10}SO_3H_2$	330.9492	7.37
$C_7F_{15}H$	368.9762	9.49
$C_6F_{13}H$	318.9798	9.03
C_3F_7H	168.9894	8.60
C_2F_5H	118.9926	1.05
$C_5F_{11}H$	268.9830	8.43

Table S4 Integrated areas of FTIR characteristic peaks for off-gases with and without additives

Additives	None	Fe_2O_3	Fe_3O_4	CaO	$Ca(OH)_2$	Kaolinite	MgO
Relative Peak Area	34.00	21.99	16.31	15.82	21.21	31.65	20.49

Table S5 Price of additives used for contaminated soil pyrolysis

Additives	Fe_2O_3	Fe_3O_4	CaO	$Ca(OH)_2$	Kaolinite	MgO
Cost (CNY/t)	1700	1900	500	700	1900	4000

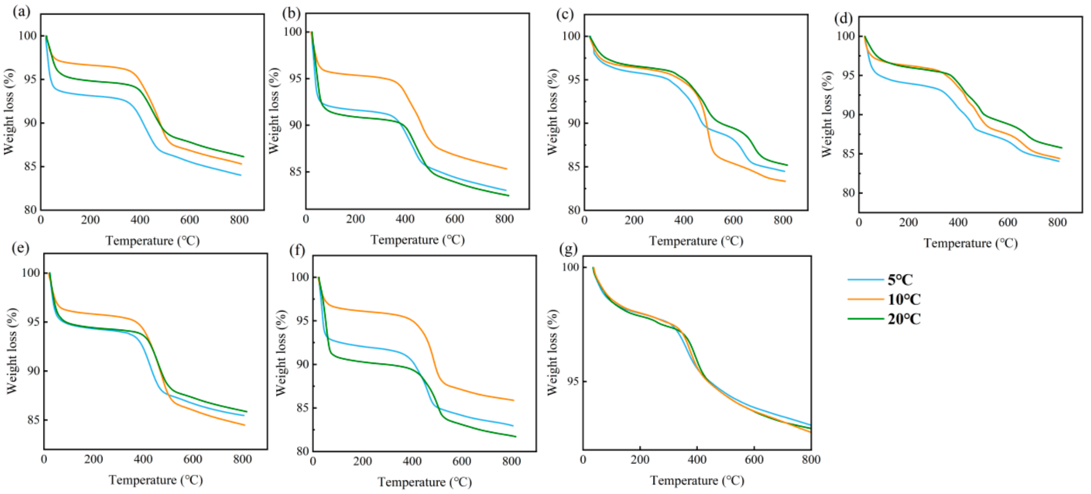

Figure S1 The thermal weight of adding different additives in PFOS contaminated soil on different heating rate. (a) Fe_2O_3, (b) Fe_3O_4, (c) CaO, (d) Ca (OH) $_2$, (e) Kaolinite, (f) MgO, (g) None additions.

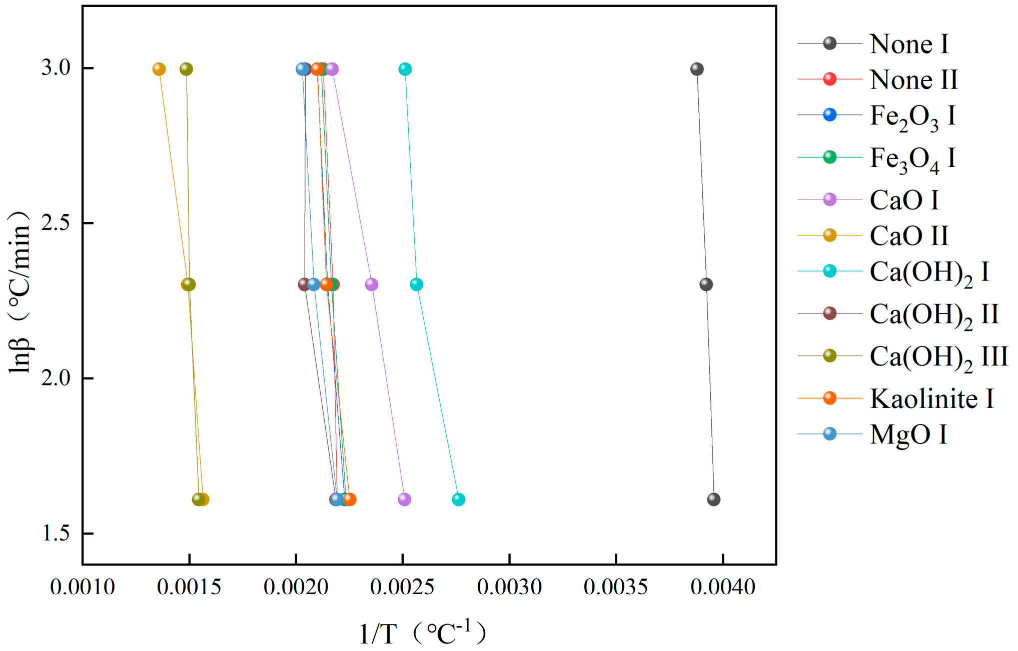

Figure S2 Arrhenius plots based on FWO method

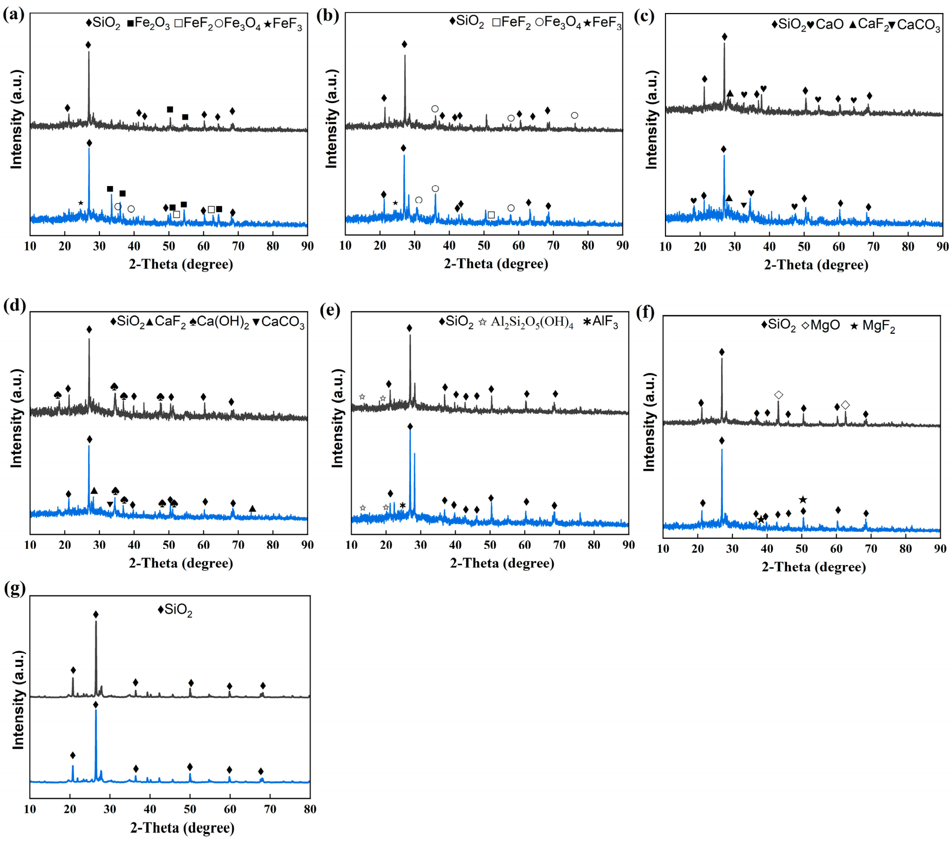

Figure S3 The XRD diagram of PFOS contaminated soil with different additives. (a) Fe_2O_3, (b) Fe_3O_4, (c) CaO, (d) Ca $(OH)_2$, (e) Kaolinite, (f) MgO, (g) None additions. Black: before heating; blue: after heating.

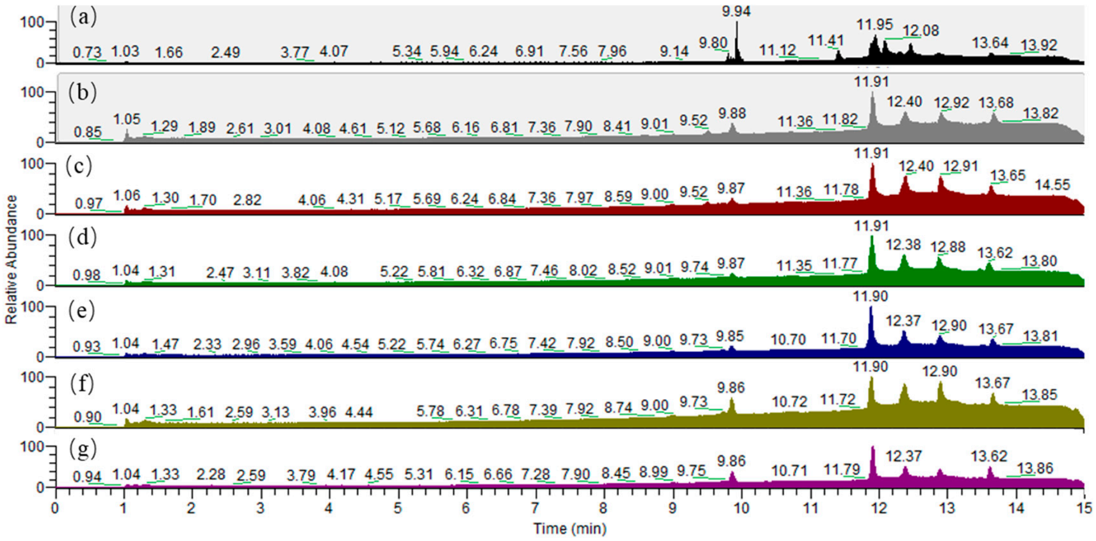

Figure S4 Chromatograms of the thermal decomposition with and without additives. (a) None additions, (b) Fe_2O_3, (c) Fe_3O_4, (d) CaO, (e) $Ca(OH)_2$, (f) Kaolinite, (g) MgO.

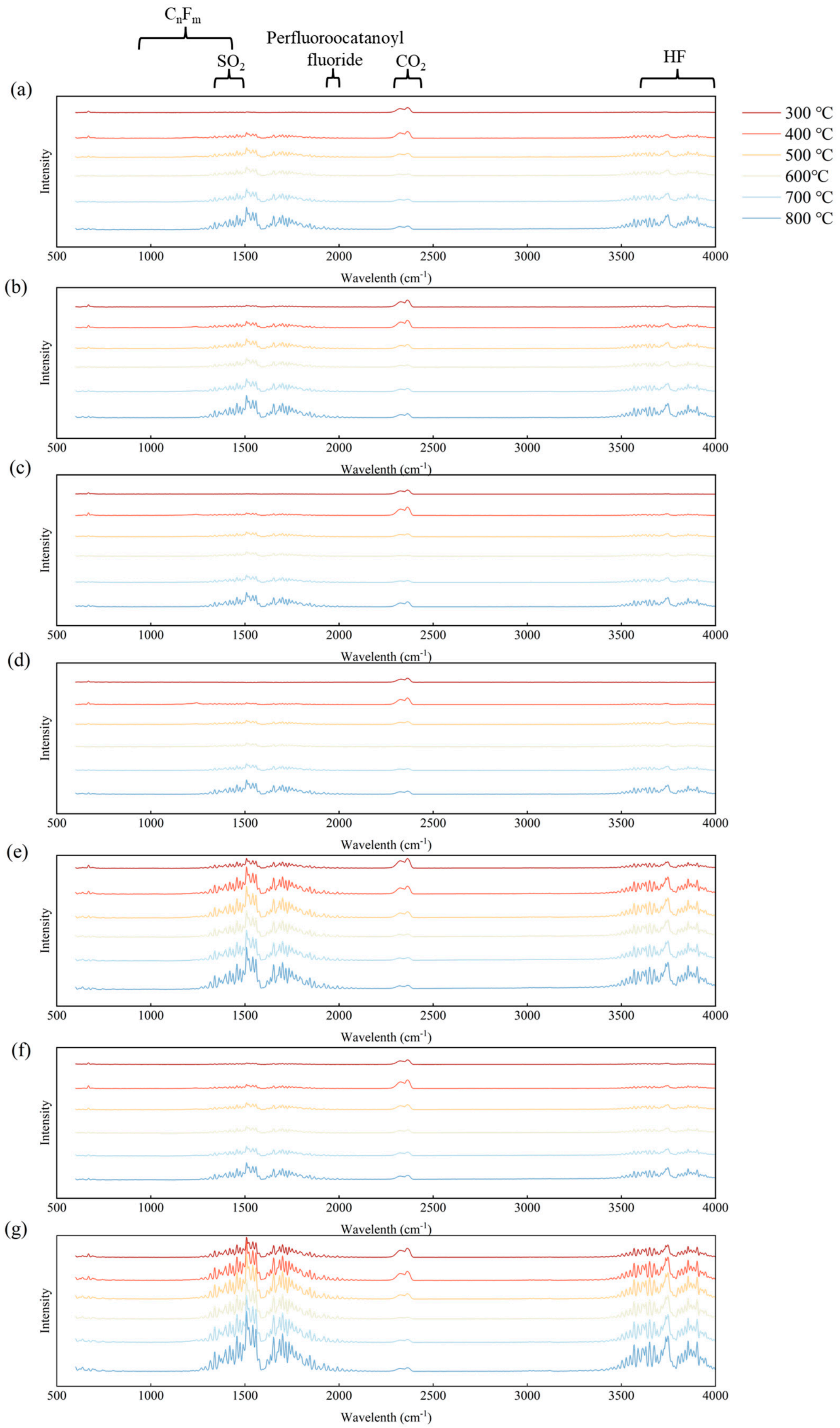

Figure S5 FTIR spectra of off-gas from thermal treatment of PFOS-contaminated soil. (a) Fe_2O_3, (b) Fe_3O_4, (c) CaO, (d) $Ca(OH)_2$, (e) Kaolinite, (f) MgO, (g) None additions.
